# Leaf Stoichiometry of *Potentilla fruticosa* Across Elevations in China’s Qilian Mountains

**DOI:** 10.3389/fpls.2022.814059

**Published:** 2022-02-24

**Authors:** Yanyan Qin, Wei Liu, Xiaofang Zhang, Jan F. Adamowski, Asim Biswas

**Affiliations:** ^1^Key Laboratory of Land Surface Process and Climate Change in Cold and Arid Regions, Northwest Institute of Eco-Environment and Resources, Chinese Academy of Sciences, Lanzhou, China; ^2^Key Laboratory of Ecohydrology of Inland River Basin, Northwest Institution of Eco-Environment and Resources, Chinese Academy of Sciences, Lanzhou, China; ^3^Qilian Mountains Eco-environment Research Center in Gansu Province, Lanzhou, China; ^4^Department of Bioresource Engineering, Faculty of Agricultural and Environmental Sciences, McGill University, Sainte-Anne-de-Bellevue, QC, Canada; ^5^School of Environmental Sciences, University of Guelph, Guelph, ON, Canada

**Keywords:** Mountainous regions, plant growth, plant adaptation, plant traits, *Potentilla*

## Abstract

As an individual plant species can develop its own leaf stoichiometry to adapt to environmental changes, this stoichiometry can provide critical information about a plant species’ growth and its potential management in the ecosystem housing it. However, leaf stoichiometry is largely undocumented in regions with large environmental changes arising from differences in elevation. The leaf stoichiometry of *Potentilla fruticosa* L., a major alpine shrub playing an important role in supporting ecosystem functions and services in China’s Qilian Mountains (Northeast Qinghai–Tibetan Plateau), was investigated at different elevations (2,400, 2,600, 2,800, 3,000, 3,200, 3,500, and 3,800 m). At each elevation, leaf elemental (C, N, and P) concentrations were measured in *P. fruticosa* leaves sampled from three plots (10 × 10 m), and edaphic properties were assessed in nine quadrats (1 × 1 m, three quadrats per plot). Temperature and precipitation were calculated using an empirical formula. Maximum and minimum leaf carbon (C) concentrations ([C]_*leaf*_) of 524 ± 5.88 and 403 ± 3.01 g kg^–1^ were measured at 2,600 and 3,500 m, respectively. Leaf nitrogen (N) concentration ([N]_*leaf*_) showed a generally increasing trend with elevation and peaked at 3,500 m (27.33 ± 0.26 g kg^–1^). Leaf phosphorus (P) concentration ([P]_*leaf*_) varied slightly from 2,400 to 3,200 m and then dropped to a minimum (0.60 ± 0.10 g kg^–1^) at 3800 m. The [C]_*leaf*_:[N]_*leaf*_, [C]_*leaf*_:[P]_*leaf*_, and [N]_*leaf*_:[P]_*leaf*_ varied little from 2,400 to 3,000 m but fluctuated somewhat at higher elevations. The main factors affecting *P. fruticosa* leaf stoichiometry were soil organic C, pH, and soil total P, and the main limiting element for the growth of *P. fruticosa* in the study area was P. In conclusion, changes in elevation affected leaf stoichiometry of *P. fruticosa* mainly due to altered soil properties, and addressing phosphorus limitation, especially at higher elevations mainly due to losses caused by high precipitation and sparse vegetation, is a key measure to promote *P. fruticosa* growth in this region.

## Introduction

A vigorous, floriferous, deciduous shrub of high genetic diversity, the widely distributed rosaceous shrub *Potentilla fruticosa* L. (a.k.a. shrubby *Potentilla* or shrubby cinquefoil), supports many ecosystem functions in the world’s colder habitats, which makes it particularly sensitive to global warming (Elkington, 1969; Miliauskas et al., 2010; Shimono et al., 2010; Yuichiro et al., 2010). Its chemical composition (Ganenko et al., 1988; Zeng et al., 2019), edibility and cosmetic properties (Nkiliza, 1999; Liu et al., 2016), antioxidant content (Miliauskas et al., 2010; Luo et al., 2016), and effects on the expression of key enzymes and hormones of glucose and lipid metabolism in rats (Yan et al., 2019) have been documented. *P. fruticosa’s* nutritive value has been shown to be influenced by grazing (Yao et al., 2019). Its leaf morphology, physiological and biochemical characteristics have been found to be altered by atmospheric pollution and soil salinity (NaCl and Na_2_SO_4_) (Liu et al., 2013; Lugovskaya et al., 2018).

However, the leaf stoichiometry of *P. fruticosa* at high elevations on the Qinghai–Tibetan Plateau (QTP) remains relatively undocumented. Leaf stoichiometry can reflect the balance and limitations in the uptake of plant macronutrients (C, N, P) that influence plants’ growth rate and life history strategies (Baxter and Dilkes, 2012; Zhu et al., 2020) and global C, N, P biogeochemical cycles (Moe et al., 2005; Liu et al., 2018). Leaf stoichiometry information is critical developing an understanding of nutrient cycling processes, in developing biogeochemical models, and in predicting plant responses to global climate change (Zhao et al., 2014, 2018). Previous studies have shown that besides disturbances such as increased CO_2_, N, and P availability (Esmeijer-Liu et al., 2009; Scott et al., 2013) and grazing (Bai et al., 2012), topographic factors such as slope, aspect, and elevation (e.g., Qin et al., 2019; Cao et al., 2020) can also affect leaf stoichiometry. This occurs through their influence on soil formation (Jenny, 1941), water distribution, and microclimate. However, topographic factors are rarely considered in leaf stoichiometry, especially for individual species.

This study’s overall objective was to examine the effects of elevation (from 2,400 to 3,800 m) on leaf stoichiometry of *P. fruticosa*, a major alpine shrub. The study was conducted in the Qilian Mountains of the Qinghai–Tibetan Plateau (QTP), the world’s highest elevation plateau. Since mean annual precipitation (MAP), mean annual temperature (MAT), and soil properties vary with elevation (Table 1), our hypothesis was that leaf stoichiometry of *P. fruticosa* would vary with elevation, and, based on Cao et al. (2020), that P would be a limiting nutrient for *P. fruticosa* growth.

**TABLE 1:** Effects of elevation on soil properties and leaf stoichiometry of *P. fruticose* (*n* = 9).

Parameters	Elevation (fixed effect)			*R*^2^ [Fixed effect and random effect (plot)]	*df*
	*F*	*P*	*R* ^2^		
SOC	109.77	**<0.001**	0.94	0.95	6
STN	102.62	**<0.001**	0.94	0.96	6
STP	4.15	**0.013**	0.33	0.39	6
SOC:STN	5.20	**0.005**	0.44	0.60	6
SOC:STP	10.73	**<0.001**	0.51	0.51	6
STN:STP	10.70	**<0.001**	0.51	0.51	6
pH	328.46	**<0.001**	0.98	0.99	6
[*C*]_*leaf*_	39.33	**<0.001**	0.79	0.79	6
[*N*]_*leaf*_	358.35	**<0.001**	0.98	0.99	6
[*P*]_*leaf*_	1.94	0.143	0.16	0.16	6
[*C*]_*leaf*_:[*N*]_*leaf*_	110.969	**<0.001**	0.92	0.93	6
[*C*]_*leaf*_:[*P*]_*leaf*_	1.13	0.396	0.10	0.10	6
[*N*]_*leaf*_:[*P*]_*leaf*_	2.11	0.118	0.17	0.17	6

*SOC, soil organic carbon; STN, soil total nitrogen; STP, soil total phosphorus; [C]_leaf_, leaf carbon (C) concentration; [N]_leaf_, leaf nitrogen (N) concentration; [P]_leaf_, leaf phosphorus (P) concentration.*

*Significant p values (p < 0.05) are in bold.*

## Materials and Methods

### Study Area

With a mean elevation of 4,000 m (closer to 3,000 m in the northeast), mean annual precipitation (MAP) of 400 mm, and mean annual temperature (MAT) below −4°C, the Qinghai–Tibetan Plateau covers 2.5 × 10^6^km^2^. Ranging in elevation from 2,200 to 5,500 m, and located in the northeastern portion of the QTP, the Qilian Mountains present two main slope aspects: south-facing and north-facing. On the south-facing slope aspects, grasslands growing on sandy-textured Kastanozem are the dominant vegetation type, whereas on the north-facing slope aspects, Qinghai spruce (*Picea crassifolia* Kom.), growing on silty-sand-textured Podzol, is the dominant species (Qin et al., 2016).

### Field Sampling

**FIGURE 1:**
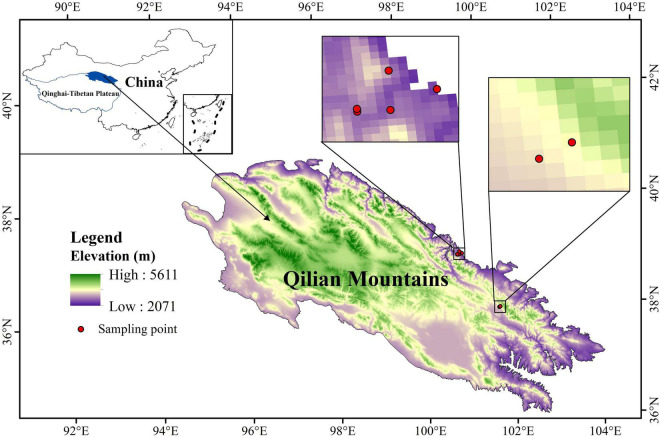
Sampling sites at different elevations (2,400, 2,600, 2,800, 3,000, and 3,200, 3,500, and 3,800 m) in the study area.

In August and September 2018, when most plant species were at the late flowering or fruiting stages, leaves from top and middle of *P. fruticosa* plants were sampled from three random 10 × 10 m plots situated at each of seven elevations: 2,400, 2,600, 2,800, 3,000, 3,200, 3,500, and 3,800 m (Figure 1). In each plot, multiple soil samples were collected from three quadrats (1 × 1 m) along the diagonal transect. A 70-mm diameter soil drill was used to sample from 3 depth profiles (0–10, 10–20, and 20–40 cm).

### Laboratory Analyses

The leaves were dried and ground to determine leaf carbon (C) concentration ([C]_*leaf*_), leaf nitrogen (N) concentration ([N]_*leaf*_), and leaf phosphorus (P) concentration ([P]_*leaf*_). Soil samples were air-dried and ground to pass through a 100-mesh sieve prior to the analysis of soil properties. A volumetric potassium dichromate method was used to determine [C]_*leaf*_ and soil organic carbon (SOC) (Nelson and Sommers, 1982), and a SmartChen 200 (AMS Rome, Italy) element analyzer was used to measure [N]_*leaf*_, [P]_*leaf*_, soil total nitrogen (STN), and soil total phosphorus (STP). A standard pH meter was used to determine soil pH in a slurry of 2.5:1 water to air-dried soil ratio (Cao et al., 2018).

### Data Analysis

The MAT and MAP (Table 1) were based on Zhao et al. (2005, 2006). For the Qilian Mountain region, these were calculated as:


(1) $$\begin{array}{c} \begin{array}{cc} MAT=20.96-5.49\times 10^{-3}ELEV-0.17LAT+8.9\times 10^{-3}LONG & \end{array}\\ R^{2}=0.98 \end{array}$$

(2) $$\begin{array}{cc} MAP=1.68\times 10^{3}+0.12ELEV+12.41LAT-75.26LONG\;R^{2}=0.92 & \end{array}$$

where MAT = mean annual temperature (°C), MAP = mean annual precipitation (mm), ELEV = elevation (m), LAT = latitude (°), and LONG = longitude (°). *R*^2^ indicates the coefficient of determination.

All data were expressed as mean and standard error (SE). The fixed effect (elevation) and random effect (plot) on soil properties and leaf ecological stoichiometry of *P. fruticosa* were tested by fitting generalized linear mixed models (GLMMs). The *t*-test was used to check the significant difference (*p* < 0.05) of each measured parameter between different elevations. Redundancy analysis (RDA) was performed to find the dominant environmental variables influencing leaf stoichiometry of *P. fruticosa* (Maccherini et al., 2011; Yang et al., 2018a). The significance of the eigenvalues of the canonical axes was tested by a reduced Monte Carlo model with 270 unrestricted permutations (Sun et al., 2017; Yuan, 2017). The Pearson’s correlation coefficient was used to determine the correlation between leaf stoichiometry and abiotic factors. SPSS 22.0 for Windows (SPSS, Inc., Chicago, IL, United States) was used to determine the Pearson’s correlation coefficient. Other analyses were performed in R4.1.1 (*vegan* and *nlme* packages). The figures were prepared using Origin 2021 (OriginLab Corp, Roundhouse Plaza, Northampton, MA, United States) and R4.1.1 (*ggplot2* and *ggrepel* packages).

## Results

### Effects of Elevation on Soil Properties and Leaf Stoichiometry of *Potentilla fruticosa*

The results from GLMMs showed that except [P]_*leaf*_, [C]_*leaf*_:[P]_*leaf*_, and [N]_*leaf*_:[P]_*leaf*_, soil properties and other elemental stoichiometries of *P. fruticosa* were significantly affected by elevation (Table 1). Compared to elevations below 3,000 m, SOC and STN significantly increased (3- to 4-fold) at elevations of 3,200 m and above. However, STP did not show this trend, as it peaked at 2,600 m (0.58 ± 0.02 g kg^–1^). Variation in SOC:STN across elevations was minimal, with the largest value (11.22 ± 0.10) occurring at 3,500 m and the smallest (8.96 ± 0.46) at 2,600 m. For SOC:STP and STN:STP, the largest and the smallest values were at 3,500 m (487 ± 133 and 42.74 ± 11.46, respectively) and 2,600 m (20.90 ± 0.67 and 2.43 ± 0.12, respectively), respectively. Soil pH values shifted from 6.12 ± 0.03 at 3,500 m to 8.54 ± 0.02 at 2,600 m. The MAT showed a decreasing trend from 2,400 to 3,800 m, whereas MAP showed a converse trend (Table 2).

**TABLE 2:** Changes of temperature, precipitation, and soil properties at different elevations (mean ± standard error, *n* = 9).

Parameter	Elevation						
	2,400 m	2,600 m	2,800 m	3,000 m	3,200 m	3,500 m	3,800 m
SOC (g kg^–1^)	13.12 ± 0.73 e	12.05 ± 0.52 e	15.08 ± 0.63 d	12.28 ± 0.61 e	35.60 ± 3.19 c	58.64 ± 1.65 a	50.14 ± 1.65 b
STN (g kg^–1^)	1.46 ± 0.08 de	1.41 ± 0.08 de	1.62 ± 0.09 d	1.35 ± 0.10 e	3.48 ± 0.23 c	5.24 ± 0.13 a	4.72 ± 0.13 b
STP (g kg^–1^)	0.53 ± 0.02 a	0.58 ± 0.02 a	0.44 ± 0.02 ab	0.32 ± 0.03 b	0.53 ± 0.03 a	0.27 ± 0.05 b	0.54 ± 0.12 a
SOC:STN	9.01 ± 0.19 c	8.96 ± 0.46 c	9.40 ± 0.27 bc	9.24 ± 0.35 c	10.09 ± 0.32 ab	11.22 ± 0.10 a	10.61 ± 0.23 a
SOC:STP	26.03 ± 2.27 d	20.90 ± 0.67 d	35.48 ± 1.50 c	41.29 ± 4.03 c	67.50 ± 3.43 b	487 ± 133.00 a	183 ± 32.61 b
STN:STP	2.89 ± 0.24 d	2.43 ± 0.12 d	3.77 ± 0.14c	4.46 ± 0.36 c	6.69 ± 0.26 b	42.74 ± 11.46 a	17.32 ± 3.18 b
pH	8.43 ± 0.04 b	8.54 ± 0.02 a	8.36 ± 0.04 b	8.51 ± 0.03 a	8.08 ± 0.04 c	6.44 ± 0.05 d	6.12 ± 0.03 e
LONG	100°21′55″	100°17′3″	100°14′28″	100°14′26″	100°22′35″	101°21′0″	101°22′12″
LAT	38°37′5″	38°33′17″	38°33′9″	38°33′22″	38°38′15″	37°40′48″	37°41′24″
MAT (°C)	2.26	1.17	0.08	−1.02	−2.13	−3.61	−5.26
MAP (mm)	304.71	331.32	355.7	379.22	398.58	518.42	553.62

*SOC, soil organic carbon; STN, soil total nitrogen; STP, soil total phosphorus; LONG, longitude; LAT, latitude; MAT, mean annual temperature; MAP, mean annual precipitation.*

*Row-wise non-matching letters indicate a significant difference among elevations (p < 0.05).*

**FIGURE 2:**
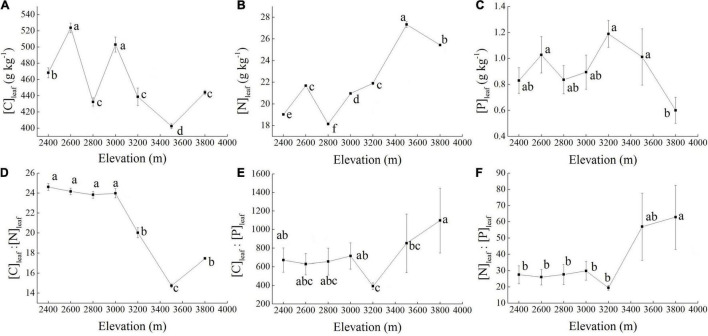
*Potentilla fruticosa* [C]_*leaf*_ **(A)**, [N]_*leaf*_ **(B)**, and [P]_*leaf*_ **(C)** and [C]_*leaf*_:[N]_*leaf*_ **(D)**, [C]_*leaf*_:[P]_*leaf*_ **(E)**, and [N]_*leaf*_:[P]l_*eaf*_ **(F)** ratios from 2,400 to 3,800 m (*n* = 9). [C]_*leaf*_, leaf carbon (C) concentration; [N]_*leaf*_, leaf nitrogen (N) concentration; [P]_*leaf*_, leaf phosphorus (P) concentration. Data are described by their mean and standard error (SE). Different lowercase letters indicate a significant difference among elevations at *p* < 0.05.

At 3,500 m, [C]_*leaf*_ (403 ± 3.01g kg^–1^) was significantly lower than that at any other elevation, whereas at 2,600 m [C]_*leaf*_ (524 ± 5.88 g kg^–1^) was significantly greater than at any other elevations except 3,000 m (Figure 2A). The [N]_*leaf*_ showed an increasing trend with increasing elevation. At 3,500 m, [N]_*leaf*_ (27.33 ± 0.26 g kg^–1^) was significantly greater than at other elevations, whereas [N]_*leaf*_ at 2,800 m (18.15 ± 0.10 g kg^–1^) was significantly lower than that at any other elevations (Figure 2B). The [P]_*leaf*_ changed slightly at elevations between 2,400 and 3,200 m and had a decreasing trend at elevations from 3,500 to 3,800 m, with the lowest value (0.60 ± 0.10 g kg^–1^) recorded at 3,800 m, which was significantly lower than that at 2,600, 3,200, or 3,500 m (Figure 2C). From 2,400 to 3,000 m, [C]_*leaf*_:[N]_*leaf*_ varied little, but increased significantly at or above 3,200 m. However, from 3,200 to 3,800 m, [C]_*leaf*_:[N]_*leaf*_ showed a decreasing and then an increasing trend, with the value at 3,500 m (14.74 ± 0.19) being significantly lower than that at other elevations (Figure 2D). Changes in [C]_*leaf*_:[P]_*leaf*_ and [N]_*leaf*_:[P]_*leaf*_ along the elevation gradients were similar. Both of them varied slightly between 2,400 and 3,000 m; however, from 3,000 to 3,800 m, their values first decreased and then increased and reached minimums and maximums at 3,200 (392 ± 35.28 and 19.43 ± 1.50, respectively) and 3,800 m (1097 ± 349 and 62.79 ± 19.81, respectively), respectively (Figures 2E,F).

### Dominant Factors Influencing Leaf Stoichiometry of *Potentilla fruticosa* at Different Elevations

**FIGURE 3:**
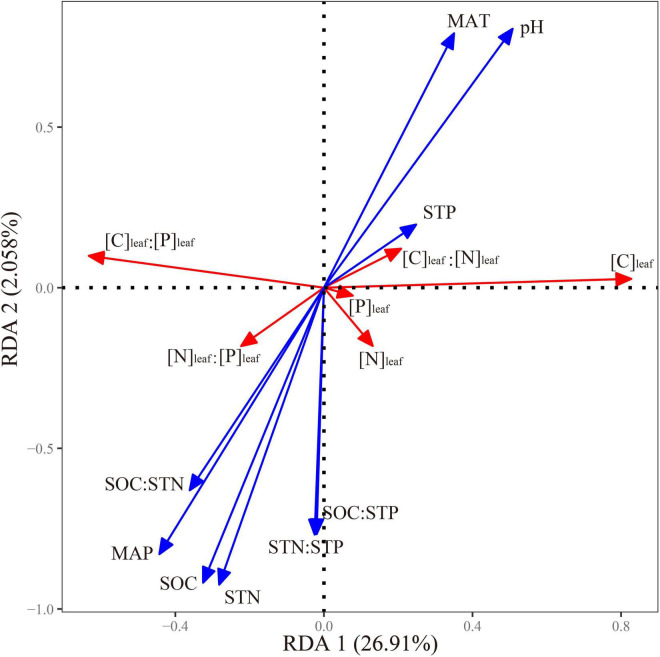
Redundancy analysis (RDA) ordination for the leaf stoichiometric indices of *P. fruticosa* and environmental characteristics. SOC, soil organic carbon; STN, soil total nitrogen; STP, soil total phosphorus; [C]_*leaf*_, leaf carbon (C) concentration; [N]_*leaf*_, leaf nitrogen (N) concentration; [P]_*leaf*_, leaf phosphorus (P) concentration; MAT, mean annual temperature; MAP, mean annual precipitation.

The RDA results showed that the eigenvalues of the first and second axes were 0.27 and 0.02, respectively (Figure 3), indicating that the two axes could explain about 29.00% of the total variation in leaf stoichiometry of *P. fruticosa* across elevations. Based on the RDA result, SOC, STP, and pH had significant effects on this variation, and they could explain 7.10, 6.80, and 6.00% of it, respectively (Table 3).

**TABLE 3:** Relationships between environmental factors and the two RDA axes, and also environmental factors that are significant contributors to leaf stoichiometry of *P. fruticosa* across elevations.

Environmental factors	RDA1	RDA2	*R* ^2^	P*	Explains (%)	*F*	P**
SOC	–0.362	–0.932	0.85	**0.001**	6.00	4.20	**0.034**
STN	–0.350	–0.937	0.84	**0.001**	3.30	2.20	0.130
STP	0.514	0.858	0.07	0.097	6.80	5.40	**0.018**
SOC:STN	–0.408	–0.913	0.47	**0.001**	2.60	1.80	0.176
SOC:STP	–0.275	–0.962	0.48	**0.001**	3.30	2.40	0.100
STN:STP	–0.277	–0.961	0.49	**0.001**	< 0.10	< 0.10	0.800
pH	0.419	0.908	0.80	**0.001**	7.10	4.60	**0.024**
MAT (°C)	0.382	0.924	0.68	**0.001**	0.50	0.40	0.532
MAP (mm)	–0.401	–0.916	0.79	**0.001**	0.50	0.40	0.506

*SOC, soil organic carbon; STN, soil total nitrogen; STP, soil total phosphorus; [C]_leaf_, leaf carbon (C) concentration; [N]_leaf_, leaf nitrogen (N) concentration; [P]_leaf_, leaf phosphorus (P) concentration; MAT, mean annual temperature; MAP, mean annual precipitation. Significant p-values (p < 0.05) are in bold.*

“*” indicates the environmental factor was significantly related to the two axes from the RDA.

“**” indicates the variance of each environmental factor significantly contributed to the total variance.

Soil pH was positively related to [C]_*leaf*_ and [C]_*leaf*_:[N]_*leaf*_ and negatively with [N]_*leaf*_, [C]_*leaf*_:[P]_*leaf*_, and [N]_*leaf*_:[P]_*leaf*_ (Table 4). In contrast, the relationship between SOC and leaf stoichiometry was the opposite of those of soil pH, except that there was no significant relationship between SOC and [C]_*leaf*_:[P]_*leaf*_. The STP was not related to any index of leaf stoichiometry.

**TABLE 4:** Relationships between leaf stoichiometry of *P. fruticosa* and the dominant factors from the RDA.

Leaf stoichiometry	SOC	STP	pH
[*C*]_*leaf*_	−.639**	0.175	.562**
[*N*]_*leaf*_	.869**	–0.216	−.867**
[*P*]_*leaf*_	–0.055	0.045	0.167
[*C*]_*leaf*_:[*N*]_*leaf*_	−.929**	0.206	.882**
[*C*]_*leaf*_:[*P*]_*leaf*_	0.199	–0.192	−.263*
[*N*]_*leaf*_:[*P*]_*leaf*_	.367**	–0.216	−.416**

*SOC, soil organic carbon; STN, soil total nitrogen; STP, soil total phosphorus; [C]_leaf_, leaf carbon (C) concentration; [N]_leaf_, leaf nitrogen (N) concentration; [P]_leaf_, leaf phosphorus (P) concentration.*

“*” p < 0.05, and “**”p < 0.01.

## Discussion

### Reasons for Variation in Soil Properties and Leaf Stoichiometry of *Potentilla fruticosa* With Elevation

By mainly influencing solar radiation and condensation of water vapor (Sevruk, 1997; Ohmura, 2012), elevation regulates temperature and precipitation (Lozano-García et al., 2016; Zhu et al., 2019), which in turn exerts effects on the distribution of vegetation. For example, in the Qilian Mountains, temperature decreases and precipitation increases with increasing elevation (Chang et al., 2014), resulting in shifting vegetation types: <2,400 m, steppe desert; 2,400–3,300 m, forest steppe; 3,300–3,600 m, subalpine scrub and grassland; 3,600–3,900 m, alpine scrubs and meadow; >3,900 m, ice and snow (Zhu et al., 2019). Likewise, different elevations differ in vegetation types, biomass, quantity and quality of litter, roots, and soil microbial communities (Bargali et al., 2018; Yang et al., 2018b), which in turn affect soil physical and chemical properties (Tables 1, 2; Zhou et al., 2013; Qin et al., 2019). In addition, microlandforms such as slope aspect, slope position, and slope gradient can also influence soil properties by reshaping hydrothermal conditions and patterns in the movement of the material and energy (Måren et al., 2015; Nabiollahi et al., 2018; Zhang et al., 2020) as found in this study (data unpublished).

With changes of biotic and abiotic environments with elevation, leaf stoichiometry of *P. fruticosa* also varied with elevation (Figure 2), concurring with other studies (e.g., Badano et al., 2005; Zhang et al., 2019). However, only [C]_*leaf*_, [N]_*leaf*_, and [C]_*leaf*_:[N]_*leaf*_ of *P. fruticosa* were significantly affected by elevation (Table 1), partly supporting our hypothesis that the leaf stoichiometry of *P. fruticosa* would vary with elevation. In contrast, Cao et al. (2020) found that except for [N]_*leaf*_:[P]_*leaf*_, leaf stoichiometries of *Oxytropis ochrocephala* Bunge in the Qilian Mountains were significantly affected by elevation. This suggests that each species may have its unique strategies to adapt to local environmental changes.

From 2,400 to 3,800 m, [C]_*leaf*_ of *P. fruticosa* showed a decreasing trend (Figure 2A), which was in contrast to Zhao et al. (2014) and Rong et al. (2016), who found that [C]_*leaf*_ increased with decreasing temperature to balance the osmotic pressure of cells and resist freezing. This result may reflect the fact that low temperatures inhibit photosynthesis in *P. fruticosa*. In contrast, [N]_*leaf*_ of *P. fruticosa* showed an increasing trend with a decrease in temperature (Figure 2B), as reported by others (e.g., Oleksyn and Przybyl, 1987; Cao et al., 2020). This may be because [N]_*leaf*_ can enhance metabolic activity and the growth rate of tissues in cold habitats and short growing seasons (Ågren, 2008; Zhang et al., 2017). With a [C]_*leaf*_ decrease and [N]_*leaf*_ increase, *P. fruticosa* [C]_*leaf*_:[N]_*leaf*_ decreased with rising elevation (Figure 2D). Similar observations were reported by Sun et al. (2017). Generally, [C]_*leaf*_:[N]_*leaf*_ reflects a plant’s ability to simultaneously absorb C and N, and a low value can benefit plant growth (He et al., 2008; Yan et al., 2015).

### The Dominant Environmental Factors Influencing Leaf Stoichiometry of *Potentilla fruticosa*

Based on RDA (Figure 3 and Table 3), it is clear that SOC, STP, and pH had a greater effect on leaf stoichiometry of *P. fruticosa* than temperature or precipitation in the Qilian Mountains. This is in slight contradiction with other studies (Sardans et al., 2011; Zhang et al., 2012a; Cao et al., 2020). For example, Cao et al. (2020) found that, across various elevations in the Qilian Mountains, temperature significantly affected leaf stoichiometry of *O. ochrocephala*, as it could dictate or control nutrient availability in soils, root absorption, and the plant nutrient budget (Reich and Oleksyn, 2004; Isles et al., 2017; Liu et al., 2019). Likewise, Zhang et al. (2012a) found that temperature and precipitation directly affected the spatial patterns of leaf elemental stoichiometry across China, as precipitation regulates the mobilization of soil nutrients (Müller et al., 2017).

Although SOC, STP, and pH were the main contributors to differences in leaf stoichiometry of *P. fruticosa*, STP was not related to any index of leaf stoichiometry (Table 4), suggesting that it had a synthetic effect on leaf stoichiometry of *P. fruticosa* in the Qilian Mountains, but this needs further study. Except [P]_*leaf*_, leaf nutrient concentrations and their ratios were all significantly related to SOC or pH or both (Table 4). The SOC was negatively related to [C]_*leaf*_, which was not consistent with Niu et al. (2016) who found that these were positively correlated because the C in leaves can enter the soil through litter. In this study, elevations ≥3,200 m had greater SOC but lower temperatures (Table 1), which limited photosynthesis and thus resulted in lower [C]_*leaf*_ (Figure 2A). This suggests that the relationship between [C]_*leaf*_ of *P. fruticosa* and SOC in the Qilian Mountains may not represent a true causality. This may also suggest that the C in soil is the structural basis for plants (Schade et al., 2003; Liu et al., 2011) as less C is captured from the atmosphere by leaves subjected to low temperatures. The SOC was positively related to [N]_*leaf*_ of *P. fruticosa*, because SOC from amino acid metabolism contains N and it can be transferred from soil to plants by the process of nutrient cycling (Delgado-Baquerizo et al., 2015; Zhang et al., 2019). Given the positive relationship between SOC and [N]_*leaf*_, there exists a negative or positive relationship with [C]_*leaf*_:[N]_*leaf*_, or [N]_*leaf*_:[P]_*leaf*_ (Table 4). Generally, SOC and pH are negatively correlated, as acidic soil is beneficial to the adsorption of organic C (Zhang et al., 2012b; Hobara et al., 2016). Therefore, the relationships between pH and leaf stoichiometry of *P. fruticosa* were converse to relationships between SOC and leaf stoichiometry (Table 4).

In this study, the measured parameters can only explain about 30.00% of the total variation of leaf stoichiometry of *P. fruticosa* (Figure 3), indicating that other factors, such as plant community composition (Wang and Moore, 2014; Zhang et al., 2019), may also control the variations. As the plant community in the study area changed with elevation, effects of intra- and interspecies competitions on leaf stoichiometry of *P. fruticosa* should also be considered to achieve a comprehensive understanding.

### Limiting Nutrients for *Potentilla fruticosa* Across Elevations

It is well known that [N]_*leaf*_:[P]_*leaf*_ rather than [N]_*leaf*_ or [P]_*leaf*_ individually can provide a better assessment of a plant’s nutrient limitations (Li et al., 2018) although this assessment is still debated. According to Soudzilovskaia et al. (2005), the growth of alpine vascular plants is limited first by N, then by P, with a mean foliar N:P mass ratio of 29 in their study area. In this study, the [N]_*leaf*_:[P]_*leaf*_ of *P. fruticosa* at 3,000, 3,500, and 3,800 m were all >29 (Figure 2F). Following the criteria provided by Soudzilovskaia et al. (2005), our results suggest that P limited the growth of *P. fruticosa* at higher elevations. Soil P deficiency is common across China (Han et al., 2005; Zhao et al., 2016), including across the entire QTP (Niu et al., 2016) and the Qilian Mountains (Xu et al., 2018, 2019; Zhang et al., 2019; Cao et al., 2020). Furthermore, at elevations ≥3,500 m, the [N]_*leaf*_:[P]_*leaf*_ was >50 (Figure 2F), suggesting that *P. fruticosa* growth was greatly restricted by available P. It is well known that in the Qilian Mountains, soil surface coverage by vegetation decreases as elevation increases. In combination with P leaching through the soil profile (Chardon and Schoumans, 2007), the lack of vegetative cover at high elevations can easily increase P losses through erosion and surface run-off (Nest et al., 2014) and can make P scarcer. However, Reich and Oleksyn (2004) concluded that plant growth in high elevations was more limited by N. This suggests that limitation of nutrient elements for plants is dependent on region.

## Conclusion

In the Qilian Mountains of the northeast QTP, soil properties were more sensitive to elevations (ranging from 2,400 to 3,800 m) than leaf stoichiometry of *P. fruticosa*. From low to high elevation, [C]_*leaf*_ and [P]_*leaf*_ decreased, whereas [N]_*leaf*_ of *P. fruticosa* increased, as an adaptation for maintaining metabolic activity in cold habitats.

Elevation only affected *P. fruticosa* [C]_*leaf*_, [N]_*leaf*_, and [C]_*leaf*_:[N]_*leaf*_, mainly through its effects on SOC, STP, and pH. Although [N]_*leaf*_:[P]_*leaf*_ of *P. fruticosa* was not influenced by elevation, its value across all elevations was relatively large. This suggests that in the study area, *P. fruticosa* growth was commonly limited by soil P, especially regarding its growth at higher elevations. As *P. fruticosa* is a major alpine shrub, reducing P losses and improving its growth conditions will play an important role in maintaining the ecologically integrated functions and services of the whole QTP.

## Data Availability Statement

The raw data supporting the conclusions of this article will be made available by the authors.

## Author Contributions

YQ: conception and design of the research. XZ and YQ: acquisition of data. WL: analysis and interpretation of data. XZ: statistical analysis. WL, XZ, and YQ: drafting the manuscript. JA and AB: revision of manuscript drafting and revision of manuscript. All authors read and approved the final manuscript.

## Conflict of Interest

The authors declare that the research was conducted in the absence of any commercial or financial relationships that could be construed as a potential conflict of interest.

## Publisher’s Note

All claims expressed in this article are solely those of the authors and do not necessarily represent those of their affiliated organizations, or those of the publisher, the editors and the reviewers. Any product that may be evaluated in this article, or claim that may be made by its manufacturer, is not guaranteed or endorsed by the publisher.
